# Cultivating Authentic Partnership: Cooperative Development of a Toolkit to Mitigate Group Harm in Biorepository‐Enabled Research

**DOI:** 10.1111/hex.70635

**Published:** 2026-03-18

**Authors:** Shauntey Kalweit, Carly Marten, Odia Kane, Kalei Glozier, David Andres, Melissa Creary, Maile M. Taualii, Joon‐Ho Yu, Megan Doerr

**Affiliations:** ^1^ Sage Bionetworks Seattle Washington USA; ^2^ Community Member (unaffiliated); ^3^ Johns Hopkins University Baltimore Maryland USA; ^4^ Michigan State University East Lansing Michigan USA; ^5^ University of Michigan Ann Arbor Michigan USA; ^6^ Hawaii Permanente Medical Group Honolulu Hawaii USA; ^7^ University of Washington Seattle Washington USA

## Abstract

**Introduction:**

Practitioners currently lack guidance on how to evaluate the potential negative impacts of biorepository research on communities. We set out to create a product for researchers and oversight boards that would assist them in ‘doing right’ by communities as they conduct this research. Given that this product would represent the interests of affected communities, it was essential that community voices led its development. One well‐documented challenge of community‐engaged research is navigating the systemic power imbalance between communities and research institutions. This paper details our participatory research (PR) approach, which sought to mitigate these dynamics through extension of the Community Engagement Studio (CES) methodology for sustained, multi‐year engagement with the same community experts and facilitators.

**Methods:**

Our adapted participatory methodology was developed and iterated through a three‐phase project. In Phase 1: Community‐Driven Conceptualisation, we convened seven 90‐min CESs (stylised focus groups) with four cohorts of community experts and facilitators from marginalised groups: American Indian, Alaska Native, Native Hawaiian, Pacific Islander; Lesbian, Gay, Bisexual, Transgender, Queer, Intersex, Asexual, Two‐Spirit; Black/African American; and individuals with high‐penetrance genetic variants. In Phase 2: Community‐Centred Design and Feedback, the community experts and facilitators reviewed the initial toolkit design generated from CES‐derived insights in six 1‐hour meetings. Finally, in Phase 3: Community Co‐Analysis, the community experts and facilitators co‐analysed toolkit pilot feedback in a series of three to five 90‐min sessions across four pilot sites. Throughout each phase, community feedback was used to drive planning, finalise materials and define takeaways.

**Results:**

Our sustained engagement promoted trust and enabled deep exploration of complex topics. Challenges included retaining community experts over time and bridging conceptual insights with concrete design.

**Conclusion:**

This paper offers a reference for optimising research impact through effective PR, highlighting the benefits of sustained engagement and strategic mitigation of power imbalances in academic‐community collaborations.

**Patient or Public Contribution:**

Members of select communities affected by group harm were included as compensated collaborators in each study phase. These community experts drove early ideation, informed beta design, co‐analysed pilot data and provided integrated feedback on this manuscript.

**Abbreviations**
CES: Community Engagement Studios
CHIRON: Community Health Interests for Researchers and Oversight Networks
IRB: institutional review board
LGBTQ+: Lesbian, Gay, Bisexual, Transgender, Queer and related communities
PR: participatory research
RTA: reflexive thematic analysis
V‐CES: Virtual Community Engagement Studios

## 1 Introduction

Biorepositories, designed to store, harmonise and render interoperable big data from myriad sources, are critical to a radically evolving health research ecosystem [1]. Despite general awareness of the risk of group harm posed by biorepository‐enabled research, there is little guidance to support consideration of group harm risks in biorepository research, planning and execution [2]. We define group harm as the potential for research findings or procedures to negatively impact a group of people, either directly or indirectly, due to their shared characteristics or affiliation [2, 3, 4, 5]. The growing movement towards biorepository‐driven research projects to address health inequities makes it all the more urgent that researchers consider the community impact of their work [6], yet including community interests in biorepository‐enabled studies is a non‐trivial undertaking [7]. As others have described, communities impacted by repository research vary in terms of internal cohesion, governance structures and self‐articulation [8]. A further complication is that this research can instantiate new communities (e.g., ‘BRCA previvors’) [2]. We set out to create a novel design product for researchers and oversight boards (e.g., ethics boards) that would help them ‘do right’ by communities in the oversight, planning, execution and reporting of biorepository‐enabled research. Given that this solution would represent the interests of affected communities on their behalf, it was essential that community voices led its development.

Participatory research (PR) approaches, which engage affected community members as partners in the research process, are increasingly recognised for producing more valid and effective outcomes than approaches that exclude community involvement [7]. PR and similar community‐oriented approaches aim to include members of impacted groups as experts by virtue of their lived experience [7, 9, 10]. However, including community members in a project does not constitute their meaningful engagement [11, 12]. One challenge to meaningful community engagement is the power imbalance of research culture, which traditionally prises academic expertise over lived experience [7, 9, 13]. When PR efforts do not account for power dynamics between academic researchers and community members, the impact of community contributions may not be realised. In such instances, the engagement effort serves no legitimate purpose, burdening community members and eroding trust [9, 14]. It is the responsibility of academic researchers conducting PR to ensure that their community engagement efforts serve as more than a ‘box‐checking’ exercise.

Community Engagement Studios (CES) is a method developed by the Meharry–Vanderbilt Community‐Engaged Research Core that aims to address power dynamics in PR by providing a replicable framework for meaningful community involvement in research projects. CES are conducted by a consistent academic support team, and CES members are compensated as community expert consultants, not research subjects [15, 16]. CES are an effective, structured approach to engaging with communities in a consultative capacity, as is its virtual counterpart, Virtual Community Engagement Studios (V‐CES) [17, 18]. Researchers have traditionally utilised CES at a single stage in a project's lifecycle. However, scholars of community‐engaged research advocate for community involvement throughout the entire research process [7, 14, 16]. Practitioners have found that sustained community engagement builds trust between parties, in turn facilitating authentic community input [13, 19, 20]. Although others have successfully iterated upon aspects of CES methodology, few have adapted CES for sustained community engagement across a project's lifecycle [17, 21, 22, 23, 24]. Especially when working with communities underrepresented in research, the quality of insights benefits when researchers allot time for relationships to grow [25, 26, 27].

This paper aims to contribute to the dialogue on sustained community engagement through the use of V‐CES for a longitudinal research and design project: Community Health Interests for Researchers & Oversight Networks (CHIRON). CHIRON is an open‐source toolkit for researchers and research oversight boards that focuses on the mitigation of group harm in biorepository research. This 3‐year project dedicated the first year to V‐CES, the second to translating V‐CES outputs into a design solution with iterative contribution of community members, and the third to piloting the toolkit with community members joining in analysis of pilot site data.

## 2 Methods

### 2.1 Ethics

Community members were engaged as expert consultants and were privy to the entire development of the toolkit; they were not research subjects; therefore, their involvement was not subject to review by an IRB [15, 28]. The protocol for the piloting of the toolkit by researchers and oversight boards, who were themselves research subjects, was reviewed and approved (WIRB #2017037); participants received information sheets and provided verbal informed consent prior to participation.

### 2.2 Recruitment

**Figure 1:**
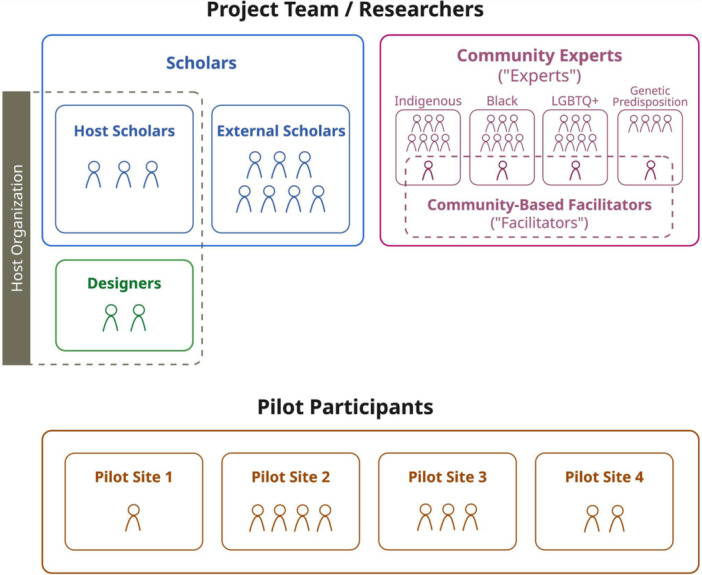
Project team and participants.

For this project, we recruited community experts and community‐based facilitators who were engaged across all three phases of the project (Table 1). In Phase 3, we recruited repository researchers and oversight board members (‘pilot participants’) to pilot the toolkit (Figure 1).

**Table 1:** Overview of communities engaged.

Communities	Number of members (experts and facilitators)		
	Phase 1	Phase 2	Phase 3
American Indian, Alaska Native, Native Hawaiian, Pacific Islander (AIANNHPI or ‘Indigenous’)	8	16	11
Lesbian, Gay, Bisexual, Transgender, Queer, Intersex, Asexual, Two‐Spirit (LGBTQIA2S+ or ‘LGBTQ+’)	8		
Black, African American, Black Caribbean, African (‘Black’)	8		
Individuals with high‐penetrance genetic variants (‘Genetic predisposition’)	5		
Total	29		

*Note:* During Phases 2 and 3, experts and facilitators were no longer segmented by community.

We recruited community experts (‘experts’)—designated as such within the CES framework, given community members' expertise in their own lived experience [16]—at the start of the project to serve as collaborators. This project sought to engage communities historically underserved in scientific research [17]. The project's scholar team brainstormed a list of those at greatest risk of being harmed by biorepository research, prioritising based on features such as intra‐community diversity and degree of formalisation (e.g., legal incorporation, existing governance) and shared identity with the project's scholars to maximise congruence. Recruitment focused on four communities: American Indian, Alaska Native, Native Hawaiian, Pacific Islander (AIANNHPI or ‘Indigenous’); Lesbian, Gay, Bisexual, Transgender, Queer, Intersex, Asexual, Two‐Spirit (LGBTQIA2S+ or ‘LGBTQ+’); Black, African American, Black Caribbean, African (‘Black’); and individuals with high‐penetrance genetic variants (‘Genetic predisposition’). Experts were recruited via snowball sampling from within scholar networks and advertisements on Twitter/X. Interested individuals followed a survey link (Qualtrics) where they could self‐identify in one or more of the focus communities. Individuals had to be 18 years or older and based in the United States. After screening and verifying eligibility, we recruited 25 experts with at least 5 years of experience in each group. Consistent with CES methodology, we also recruited community‐based facilitators (‘facilitators’; O.K., K.G. and D.A;. A.D. in Acknowledgements) for each CES group (Figure 1). Facilitators were recruited through host scholar networks and selected based on prior experience with group facilitation, shared identity with the expert groups, and availability. Experts and facilitators were compensated hourly for both synchronous and asynchronous work through all project phases.

Pilot participants, who were recruited to pilot the toolkit, reflected its target users: biorepository‐enabled researchers and oversight boards. Pilot participants were recruited using a combination of purposive and convenience sampling. After the project team established target demographics (e.g., researchers, data access committee members and IRB members), the host and academic scholars shared an overview of the pilot across their professional networks via in‐person conversation, email and social media. This yielded 16 interested groups across 10 institutions. Final participants were selected based on alignment with target demographics, ability to commit to the study length and approval of home institutions. Ultimately, four groups participated in the pilot, including repository‐enabled researchers, heads of repositories, data access committee members and human subjects research professionals.

In addition to community experts and facilitators, the project team was composed of scholars and designers employed by the host organisation (‘host scholars’ and ‘designers’), and scholars located at external institutions (‘external scholars’) (Figure 1).

### 2.3 Procedure

Communities were engaged across three project phases: (1) conceptualisation via V‐CES (Community‐Driven Conceptualisation), (2) community‐centred design (Community‐Centred Design and Feedback) and (3) piloting and co‐analysis (Community Co‐Analysis).

#### 2.3.1 Phase 1—Community‐Driven Conceptualisation

Phase 1 (June–September 2023) explored the problem space and initiated toolkit conceptualisation through V‐CES. Each group convened for 90 min on a bi‐monthly schedule for a total of 28 meetings. V‐CES meetings were hosted by the group's facilitator and an external scholar acting as a consultant. V‐CES groups used Miro [29], a virtual whiteboarding platform, to coordinate group activities. Following each meeting, host scholars and facilitators synthesised insights to inform subsequent weeks' activities. V‐CES meetings were recorded for note‐taking purposes. These recordings also became a way for experts to communicate with those not on the call, for example, ‘I want the project team to know…’ Meetings were summarised in notes, consistent with established practices [15]. V‐CES notes were shared with experts and facilitators for review and feedback. Summary notes from all 28 meetings were distilled by three facilitators and a host scholar into ‘Value Statements’ and ‘Specific Recommendations’. This documentation formed the basis for Phase 2.

On alternate weeks, the host scholars met with external scholars and facilitators to prepare for the following week's meeting with experts, creating a shared facilitator guide. Preparation also involved creating informational content in the form of short, animated videos (Animaker [30]). Videos were shared with experts in advance of V‐CES to build their knowledge base (see Appendix SA1). In addition, external scholars were provided with a short list of case studies to further frame V‐CES discussions.

A summary of methodological adaptations we made to the CES model is described in Table 2.

**Table 2:** Summary of methodological adaptations.

	**Traditional CES (Joosten et al. [** 16 **]; Israel et al. [** 15 **])**	**Virtual CES (V‐CES) (Zisman‐Ilani et al. [** 17 **]; Buell et al. [** 18 **])**	**CHIRON**
Medium	In person	Virtual (video conferencing platform)	Virtual (video conferencing platform)
Information provided	Researcher presentation often includes slides	A series of videos of researchers addressing questions	Series of animated videos illustrating a fictional case study; scholar remarks at beginning of meeting
Engagement period	One‐time engagement per study	One‐time engagement per study	Longitudinal, iterative engagement for a single study

#### 2.3.2 Phase 2—Community‐Centred Design and Feedback

During Phase 2 (December 2023–July 2024), host scholars collaborated with host organisation designers, external scholars and facilitators to synthesise V‐CES insights, finalise the toolkit's design and solicit feedback from experts in preparation for the pilot phase. At this point and moving forward, experts and facilitators were no longer segmented by community.

### 2.4 Synthesis

From December 2023 to March 2024, host scholars and designers translated the V‐CES Specific Recommendations into ‘Preliminary Design Ideas’, or tangible feature ideas. The three remaining facilitators (O.K., K.G., D.A.) and a stand‐in host scholar (C.M.) compared these ideas with their respective groups' Value Statements and Specific Recommendations to evaluate whether the Preliminary Design Ideas adequately represented their communities' intentions. Facilitators were consulted in three 60–90 min meetings, producing a final list of feasible design ideas aligned with community values.

### 2.5 Finalisation and Execution

Host scholars and designers paired final design ideas with the project's pre‐existing constraints, resulting in the finalised concept of an openly‐accessible online toolkit. As specified by the project's original proposal, the toolkit employs nudge theory, a behavioural framework used to encourage ‘positive’ behaviour change [31]. Nudging has been used to encourage a wide variety of desired behaviours, and appears to influence behaviour by targeting the cognitive biases and heuristics underlying less desired behavioural pathways [32, 33, 34]. The project team employed this framework in CHIRON to nudge repository researchers and oversight professionals toward community‐conscious practices; this constituted a departure from experts' desire for a more didactic educational approach expressed in V‐CES.

**Figure 2:**
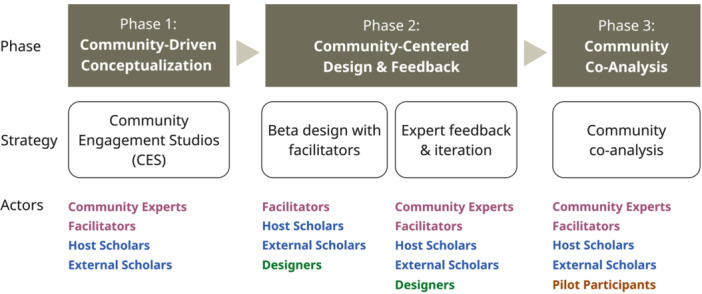
Overview of project timeline and roles.

From March to May 2024, host scholars and designers constructed a beta‐version of the toolkit website. This iterative process involved frequent review of community documentation (e.g., Value Statements) along with three additional 60–90 min feedback meetings with facilitators to ensure the toolkit's content reflected the V‐CES outputs (Figure 2).

### 2.6 Community Feedback and Iteration

To refine the beta‐version of the toolkit, host scholars and designers organised two rounds of review with experts. The full panel of V‐CES experts and facilitators were invited via email to reconvene in May 2024, with the option of attending one of three pre‐scheduled hour‐long virtual meetings or separately scheduling an individual feedback meeting. Experts were sent a 3‐min re‐orientation video about the purpose of the project and were asked to spend 1 h reviewing the website and toolkit in advance of the review meeting, making note of their impressions.

During feedback meetings, host scholars solicited feedback and posed targeted questions. Fourteen of 29 (48%) experts and facilitators attended one of the first round meetings. Updates made by host scholars and designers were presented in a second round of hour‐long meetings held in July. Ten of 29 experts and facilitators joined, with an eleventh expert providing feedback via email (11 of 29, 38%). Host scholars and designers continued to refine the toolkit through August 2024.

#### 2.6.1 Phase 3—Community Co‐Analysis

In Phase 3, CHIRON—a 10‐module online self‐paced toolkit for repository researchers and oversight boards—was piloted at four sites. Community experts were re‐engaged in co‐analysis sessions led by facilitators to assess toolkit effectiveness through evaluating data from pilot sites.

### 2.7 Pilot Context

Following IRB review and approval, four groups of researchers and oversight boards piloted the CHIRON toolkit on a rolling basis from October 2024 to June 2025; pilots ranged from 3 to 5 months in duration. All pilots were mixed‐method investigations and included one‐on‐one or group interviews. In addition, one pilot site completed a weekly survey‐style diary study. Three sites recorded their weekly explorations of the toolkit over video call.

### 2.8 Co‐Analysis

Host scholars emailed the original pool of 29 experts and facilitators, describing the planned co‐analysis tasks, time commitment and compensation to solicit interest in participation via Google Form. Nine experts and two facilitators (O.K., K.G.) (11 of 29, 38%) participated in one or more co‐analysis groups. Co‐analysis groups were held virtually, with 2–3 experts and 1–2 facilitators; the small group size enabled in‐depth discussion [35]. Co‐analysis groups were not segmented by community identity; experts were assembled according to shared availability. Host scholars organised 1‐h virtual orientations for each co‐analysis group, sharing non‐identifying context about the pilot site, guidance on tasks, privacy and confidentiality requirements and a refresher of the platform Miro. While host scholars shared the complete list of research objectives for the project, experts and facilitators were asked to focus primarily on impressions of the toolkit's impact.

Transcripts and qualitative survey responses were de‐identified by host scholars and released to co‐analysis groups in 3–4 batches. For pilots with recorded exploration, raw batches included up to 167 pages of data. To manage expert workload, a host scholar (S.K.) excerpted these batches with a focus on passages most relevant to toolkit impact. Excerpted batches contained ≤ 40 pages.

Batches of data were shared with experts on an approximately monthly cadence. Experts were given a minimum of 7 days to review batches. Before co‐analysis sessions, experts were asked to add up to five ‘quotes of interest’ to their group's Miro board. Co‐analysis sessions were led by the facilitators for 90 min. A typical session involved a brief introduction from the host scholar. Facilitators then led co‐analysis, beginning with a round robin activity to share quotes, sparking discussion that comprised the bulk of the sessions. When 15–20 min remained in the session, experts focused on collaboratively clustering quotes. Quote ‘clusters’ formed the basis of themes, to which experts gave titles and brief descriptions. A new Miro board was created for each co‐analysis session to encourage fresh interpretation, but previous boards remained accessible. Experts repeated this process of asynchronous review followed by synchronous co‐analysis sessions for each batch of data from their pilot site.

This approach to community co‐analysis borrows elements from Reflexive Thematic Analysis (RTA) [35], the method used by host scholars for analysis, and affinity diagramming, a practically‐oriented analysis practice used in User Experience research [36]. Community co‐analysis borrowed from RTA by ‘exploring, interpreting and reporting *relevant* patterns of meaning across a dataset’, [35, p. 224], with the goal of identifying shared themes. However, experts were not pushed to ensure their themes aligned with the formal qualifications for themes [35, pp. 229–230]. This approach was chosen given the time‐consuming nature of formal qualitative analysis, as well as the practical goals behind these efforts (e.g., the toolkit functioning to community expectations, identifying next steps for toolkit refinement).

In addition to recordings, each co‐analysis session produced a Miro board containing each expert's ‘quotes of interest’, as well as the group's quote ‘clusters’ with theme titles and descriptions created during affinity diagramming. Community co‐analysis outputs will be integrated with the analysis outputs of scholars to establish the findings of this project.

## 3 Results

Across this 3‐year project, experts and facilitators contributed a total of 1026 h of synchronous and asynchronous work (Tables 3 and 4). Expert hours were largely synchronous: Phase 1 included seven 90‐min meetings per expert, Phase 2 included two 1‐h meetings per expert and Phase 3 included four to seven 90‐min sessions per expert. Facilitators additionally contributed substantial asynchronous effort (Table 4).

**Table 3:** Community expert hours contributed by project phase.

Phase	Individual community expert hours	Number of community experts	Total number of community expert hours spent per phase
1: Community‐Driven Conceptualisation	12	25	300
2: Community‐Centred Design and Feedback	3	16	48
3: Community Co‐Analysis	12	11	132
Total hours contributed by community experts	480		

**Table 4:** Facilitator hours contributed by project phase.

Phase	Individual facilitator hours spent in direct engagement with experts	Individual facilitator hours spent on asynchronous planning/preparation	Number of facilitators	Total number of facilitator hours spent per phase
1: Community‐Driven Conceptualisation	10.5	11	4	86
2: Community‐Centred Design and Feedback	0	56	3	168
3: Community Co‐Analysis	16.5	55	2	143
Total hours contributed by facilitators	546			

### 3.1 Phase 1—Community‐Driven Conceptualisation

V‐CES provided meaningful insight into community perspectives on group harm from repository‐enabled research.

Facilitators and host scholars distilled V‐CES documentation into five ‘Key Insights’ per group (Table 5), which were shared back with experts for review and verification. These insights include some overlap between groups, and some may be able to be generalised more broadly.

**Table 5:** V‐CES‐derived key insights regarding community interests in repository‐enabled research.

Community type	**Key insights**
American Indian, Alaska Native, Native Hawaiian, Pacific Islander	1. Ability for re‐consent and return of samples 2. Tension between wishes of community and wishes of individuals 3. Impossibility of preventing all possible harm 4. Nothing about us without us (in leadership positions) 5. For Native Hawaiians (at minimum), research should focus on community priorities and be transparent and accountable to community
Lesbian, Gay, Bisexual, Transgender, Queer, Intersex, Asexual, two‐spirit	6. Centring the preferences of who have the most ‘skin in the game’
	7. Digital platform with trackable IDs for participants
	8. Empowerment as the primary objective of community engagement
	9. A social credit score for researchers
	10. Offering internships for community members
Black, African American, Black Caribbean, African	11. Navigating subgroups and intra‐group conflict 12. Value alignment between researchers and communities 13. Long‐term investment in community engagement 14. Community engagement should be accessible, transparent, stimulating and minimally invasive 15. List of ways to practice transparency
Individuals with high‐penetrance genetic variants	16. Researchers stating what end goal their project is in service of, beyond their specific research question 17. Tension between a false sense of urgency in research (often from funders) and a real urgency to devise treatment options 18. Requiring documentation that researchers have considered risks to groups 19. Guidance for for‐profit biorepositories 20. Entering a ‘risk dialogue’ between researchers and communities

### 3.2 Phase 2—Community‐Centred Design and Feedback

Synthesis of V‐CES summary notes led to the creation of an online, self‐reflective toolkit designed to be used by repository researchers and oversight boards.

Examples of translations from community outputs (included in Table 5) to toolkit features included:

- Key Insight 16: *Researchers stating what end goal their project is in service of, beyond their specific research question*
  Based on this insight, the toolkit asks researchers to document their immediate and overarching research goals using structured prompts. The toolkit presents a set of verbs (e.g., eliminate, identify, discover) that users must employ in their response, honing response options with the goal of making plain researchers' ultimate intent. This Key Insight arose from the genetic predisposition V‐CES; experts raised concerns about research or advocacy organisations with missions that may not align with some community members' views [37, 38]
- Key Insight 5: *Research should focus on community priorities and be transparent and accountable to the community*
  Based on this insight, the toolkit guides users to identify priority‐setting literature or grey literature (e.g., advocacy statements, calls to action) that support the priority of their research question for the community being studied. This insight was derived from the frustration that experts from the Indigenous V‐CES expressed about repository‐enabled research that uses genomics to trace the migration patterns of Indigenous peoples, studies that were seen as reflecting the curiosity of researchers rather than responsiveness to the health concerns of Indigenous communities
- Key Insight 9: *A social ‘*credit score’ *for researchers*
  Based on this insight, scholars developed a researcher self‐assessment tool called the Trustworthiness Calculator that poses closed‐set questions regarding research practices in relation to communities. It returns a numerical score corresponding to one of three levels of expertise in the practices of community orientation and awareness and recommends CHIRON toolkit resources to help researchers advance their practice. This key insight and resulting feature stem from a desire for biorepository researchers to face more accountability in cases of data mishandling, as expressed in the LGBTQ+ V‐CES.

### 3.3 Community Feedback and Iteration

As described in the *Procedure*, host scholars held two rounds of review sessions with experts to refine the beta‐version of the toolkit. Through these discussions, host scholars asked experts and facilitators for feedback on all elements of the toolkit, including resource development, clarity, appeal to users and effectiveness. Examples of feedback and consequent changes are included in Table 6.

**Table 6:** Examples of changes made based on expert feedback.

Focus	Expert feedback	Change made
Resource development	The Trustworthiness Calculator should be featured more prominently	Trustworthiness Calculator was given its own tab in the main menu
	The Trustworthiness Calculator should refer people to certain CHIRON tools based on their score	Implemented suggestion in the Trustworthiness Calculator results section
	The toolkit may be used by members of teams who lack authority over project decisions	Added question to Tool 1 asking about the respondent's role
Clarity	Some tool names do not represent the tool contents well	Changed several tool names to be more reflective of tool contents
	The Home and About pages do not explain the purpose of the toolkit	Added introductory and background content to these pages
Addressing harm	Tool 4 should give researchers more time to consider potential research harms	Moved question about potential harms to the beginning of Tool 4
	Tool 9 asks about potential harms from research and should also ask researchers how they will mitigate this	Added a follow‐up question asking researchers how they will mitigate harm
Value proposition	The website should better highlight how the tools can help with producing specific documentation	This feature was highlighted prominently on the homepage

### 3.4 Phase 3—Community Co‐Analysis

Community experts and facilitators co‐analysed the data from a given pilot site with a focus on whether or not the toolkit was having the impact they intended.

In each co‐analysis session, experts and facilitators synthesised themes from the pilot data. As an example, the following is a summary of the themes produced during one co‐analysis session (see Appendix SA1 for screenshot):

- Theme 1: Transparency—The participants wanted the toolkit to be more transparent about what would be done with the information they enter into it and what they would receive back after filling out the tool.
- Theme 2: Accessibility—The participants found the website lacking in accessibility (i.e., quantity of text, reading level, etc.)
- Theme 3: Scepticism/Defensiveness—The participants were sceptical because of the lack of transparency noted in theme 1.

Across all four co‐analysis groups, some experts and facilitators were hopeful about the impact of CHIRON, while others were doubtful.

Realised Impact

- Group 1, Session 1, Expert A:
  ‘This was a recurring theme I saw over and over…the researchers were often forgetting about the people, and CHIRON really made them re‐evaluate their thoughts…so I thought we were doing our job there, making them realise that there are people behind the data.’
- Group 2, Session 3, Expert B:
  ‘I'm watching them grow through each dataset…we're watching them, as they engage with the tools, shift their thinking and their views, which is actually literally nudge theory working.’
- Group 3, Session 3, Expert C:
  ‘The toolkit is essentially working in the way it's intended to…everything is being communicated well and they're understanding the use of the toolkit. Maybe the dismount is a little bit shaky.’

Lack of Impact

- Group 2, Session 2, Expert B:
  ‘They fundamentally misunderstood the point of the toolkit. They're looking to us to act as the authority figure for them, rather than looking to take on that authority or responsibility themselves…One is about meaningfully addressing harms, one is about feeling better about what one is doing.’
- Group 4, Session 3, Facilitator A:
  ‘This is towards the end…they might be bought into [CHIRON's] value and significance, but there seems to be a disconnect between them internalising that responsibility and wanting to act on it.’

## 4 Discussion

Our aim to develop and pilot a toolkit aimed at reducing group harm in biorepository‐enabled research necessitated a PR approach that directly channelled the intentions and perspectives of communities into the resulting solution. A central challenge in the conduct of PR is successfully navigating power dynamics between academic researchers and community‐based collaborators that threaten to undermine the quality and legitimacy of community engagement [9, 13, 39]. Our extension of the V‐CES framework for 3 years of sustained engagement with community experts and facilitators, in tandem with other specific methodological choices, was undertaken to mitigate culturally embedded power imbalance and to promote authentic community input and translation of contributions.

### 4.1 The Value of Sustained Community Engagement

Our extended approach to PR enabled us to create a ‘safe space’ for experts to share their views, owing to the rapport established amongst group members. Our experience was consistent with related literature, which highlights building mutual trust as a crucial component of community engagement [19, 20, 40] and the importance of time in this process [13, 19, 41]. Murray et al. [42] found that ‘repeated interviews give participants implicit permission to broach what was previously unspeakable, facilitating frank and honest discussions that might otherwise not have occurred’. For instance, in their final V‐CES meeting, in discussing a case example on clinical assessment algorithms [43]. Black experts suggested such tools should be tested first on White patients to ensure they were not harmful before being used on Black patients. This anecdote illustrates the depth of within‐group trust, cultivated over time, that allowed for honest and profound community engagement with the project's focus. A similar study by Valdez et al. [24] that likewise retained the same community experts for CES over multiple project phases attests to this point, noting that this approach ‘may have enhanced progression in trust‐building…and group cohesion for both participants and researchers’.

In addition to opening the door to the sharing of ‘in group’ views, the project's extended community engagement structure contributed to the richness of insights generated. Holding multiple sessions with consistent individuals, during both Phases 1 and 3, enabled meetings to build on one another, allowing for deeper exploration of complex topics and more thoroughly developed contributions [26, 27]. Corroborating this experience in the context of conducting serial interviews, Read attests, ‘it is folly to expect to obtain full “answers” all at once. Instead, a faithful and well‐rounded picture of the answer will emerge only over time.' [26] These cumulative benefits apply not only to insights but also to preparation. A known feature of inequitable community engagement is the failure of academic researchers to adequately prepare community collaborators to fully engage with the topic at hand [17, 44]. In an assessment of the CES methodology, Joosten et al. [44] found that a common theme in expert feedback was difficulty understanding researchers due to overly technical presentation of information and the desire for better informational preparation for CES meetings. While the authors' suggestion to provide accessible preparation materials is helpful, short‐term engagement inherently poses limitations on the preparation that can be provided. Literature on sequential focus groups demonstrate that sequenced meetings allow non‐researchers to build their knowledge base of the project over time, [45] a strategy that can improve knowledge retention [46].

Given that our project is centred around the niche topic of group interests in biorepository‐enabled research, community preparation was of particular importance. The project's sustained engagement structure allowed experts to build and solidify their knowledge base of the topic and project gradually over time. During Phases 1 and 2, we used a series of 2.5–7 min case study videos to build knowledge and project‐specific context. To enable meaningful involvement in co‐analysis during Phase 3, we developed a guidebook for experts (see Appendix SA1). The guidebook included straightforward instructions and examples, ensuring all experts started on equal footing. In addition, a brief practice exercise during the Phase 3 kickoff meetings provided experts opportunity for clarification and reinforcement. Our purposeful approach to engaging experts via this preparation returned insights that drove and enriched all three phases of the project.

### 4.2 Structural Choices to Improve Engagement

In alignment with Joosten et al.'s [44] CES guidance to ‘[limit] the number of researchers in the room’, we believe that decentring institutionally based researchers was a key structural choice that improved engagement quality. Strategies included having a host scholar attend V‐CES meetings only to perform administrative tasks and ensuring that scholar team members served as informational resources, not leaders. In co‐analysis sessions, a junior host scholar attended for administrative and technical purposes but did not participate in the discussion. External scholars reviewed community co‐analysis outputs asynchronously to prevent their perspectives from influencing experts' analyses.

In place of host scholars, community‐based facilitators led the majority of community expert discussions. Scholars have noted that ‘insider’ status provides an advantage in trust‐building with communities [47, 48, 49], as this entails ‘more balanced power dynamics’, [48] and as there may be ‘an assumption of understanding’ among members of the same community [47]. Ensuring that facilitators's identities were broadly congruent with their group's experts removed an element of social difference that often undermines community‐engaged research [9]. One facilitator (K.G.) noted that in addition to sharing a degree of lived experience, they and their group's experts shared ‘skin in the game’ in regard to the project's focus on group harm.

Differences in technology access were also considered. Computer ownership and internet access differ notably in association with factors such as race and income [50, 51, 52]; while planning the project's V‐CES phase, host scholars intentionally designed activities that would allow participation via mobile devices, allowing the project to benefit from a broader range of perspectives.

### 4.3 Ensuring Community Voice in Outputs

Another common manifestation of the power imbalance in PR occurs when community insights are collected but not meaningfully acted upon or incorporated into study outputs. Joosten et al.'s [44] assessment of CES found, for example, that some community experts felt that researchers did not value or listen to their input. A review of community‐engagement practices by Shim et al. [14] returned a similar result and, moreover, found that researchers themselves ‘often struggled to recall how their studies used the community input that had been collected’. Burdening communities with the ‘theatre’ of engagement can result in ‘research fatigue’ [14] and damaged relationships as community members become disillusioned with performative community‐engaged research initiatives [14, 19, 53]. Although our project included community involvement in each major phase, we did not involve experts in activities where there was no clear utility for their input [54]. Host scholars communicated transparently with experts about toolkit limitations when input could not be acted upon.

We prioritised ensuring that the insights of experts were represented in the outputs of our study, as can be seen in the results of Phases 2 and 3. For example, one feature of the final toolkit design that would not exist if not for this community‐driven process is the previously described Trustworthiness Calculator, which originated from the desires of the LGBTQ+ experts for a TrustPilot‐like tool for biorepository researchers. Concerned that a review‐based scoring tool would recapitulate bias as seen in other such tools (e.g., Rate My Professors [55]), the concept was abandoned by the host scholars. However, experts continued to emphasise the importance of this recommendation, leading to a tool that captures the intention and has elicited positive responses from both experts and participants. In addition, the expert quotes included from Phase 3 illustrate that while the toolkit was not always as effective as intended, experts recognised it as a product of the intentions and values they provided in earlier phases.

### 4.4 Centring Human Relationships Over Rigid Proceduralism

Throughout the project, host scholars sought to balance methodological rigour with flexibility and humanity, acknowledging that community‐engaged research, like all research, takes place within the context of real lives. A potent example of this was in response to the devastating fires in Maui in 2023. The fires directly affected experts from the Indigenous V‐CES. A group chat used by group members served as a tool for check‐ins and mutual support. Rather than going forward with the next V‐CES meeting's planned agenda, facilitators and host scholars opened the time as an optional drop‐in session for experts to connect with one another. Practitioners of community‐engaged research have emphasised the importance of this human‐first response to life‐disrupting events, even if it may seem to contradict traditional research practices that emphasise objective boundaries and prioritisation of the research agenda [56, 57]. Actions that challenge traditional research priorities, such as flexibility regarding study protocols, checking in with community members about their needs outside of the study and including community members in decision‐making on how to move forward, ultimately benefit a project by ‘nurturing a sense of partnership and community’ and by building trust through demonstrating trustworthiness [56, 57, 58, 59].

Throughout the project, host scholars encouraged facilitators to shape the project's approach to expert engagement. Within Phase 1, ‘off week’ meetings with facilitators and host scholars provided an opportunity for co‐creation of the V‐CES agenda and facilitator guides. In Phase 2, host scholars and facilitators iterated upon planned procedures to produce sessions that were insightful and constructive. This collaborative and iterative approach, in contrast to research practices in which the ‘preset [study] design does not change over the course of the project,’ [60] is commonly cited as a key tenet of effective community‐engaged research [19, 60, 61]. As a prominent example, Cargo et al. [7] argue that ‘The upper bound of participation occurs when…academic and nonacademic partners codirect each phase of the PR research process’.

This focus on people over procedures manifested more subtly in the project team's approach to expert compensation. Another draw of the CES methodology is the emphasis on fair compensation of experts [16], which, in addition to being ethical, has been shown to play a role in trust‐building [62]. The team took a deliberate approach to compensation that demonstrated trust in experts as valued co‐researchers, including self‐reporting of asynchronous project hours and ‘rounding up’ synchronous research activities to the nearest half hour. These practices align with the literature covering compensation in community‐engaged research, in which we have seen flexibility [56, 63], affordance of agency [64] and respectful tracking of expenditures [65] upheld as best practices.

## 5 Challenges and Limitations

### 5.1 Logistical Challenges

During initial recruitment, after posting about the study on social media, a number of ‘fake’ applicants for the expert role responded to the interest survey, a growing problem in research broadly [66, 67]. These were applicants who did not meet the criteria for the study, some of whom posed as multiple people. A host scholar (C.M.) held 5‐min screening interviews with each applicant being considered, which proved an effective but time‐intensive solution.

Logistical ‘hiccups’ occasionally delayed experts' compensation; payment systems at research organisations are not typically set up for community members to be compensated as professionals, and the host organisation was no exception [68]. These delays may have eroded trust with experts as co‐researchers.

The project saw a decrease in expert and facilitator involvement as time progressed, as is common in longitudinal research [69]. Paying experts and facilitators likely played an essential role in retaining the individuals who collaborated in later phases [58, 59]. Uneven retention across expert and facilitator identities may have resulted in some community identities receiving less representation than others.

### 5.2 Methodological Challenges

Experts struggled to consistently distinguish biorepository‐enabled research from primary data collection methods—a challenge that extended to pilot participants (i.e., repository researchers and oversight boards) as well, highlighting the complexity and novelty of big health data research broadly. While multimedia materials proved effective for promoting understanding, context confusion persisted and at times led to tangential discussions.

A concern in the practice of community‐engaged research is that community contributions are effectively discarded when academic researchers do not translate them into outcomes [9, 53]. We observed that tangential discussions could lead to suggestions that were not relevant to the topic of biorepository‐enabled research or to our specific use‐case. These ranged from infeasible inputs, such as the desire for re‐consent of existing biorepository participants, to general frustrations with failings of the US healthcare system. In some cases, a suggestion not feasible in its raw form could be translated into a solution that retains the underlying intention, for example, the aforementioned Trustworthiness Calculator, but this was not always possible.

Furthermore, host scholars and designers initially experienced a disconnect when the carefully derived Value Statements and Specific Recommendations were not concrete enough to be translated into tangible design elements. This disconnect was overcome through synchronous brainstorming and by incorporating the project's pre‐existing design constraints into the ideation process.

### 5.3 Limitations

While the authors believe that the study's findings have strong potential for transferability, this cannot be guaranteed; it is possible that had different communities among those impacted by group harm been selected, different insights may have been generated. It is also possible that had different individuals been recruited for the groups included in this project, different insights may have emerged. However, in the interest of producing broadly relevant outcomes, priority was placed on assuring that the selected communities (Indigenous, LGBTQIA2S+, Black and genetic predisposition) represented a diversity of group characteristics, including level of visibility, level of internal organisation and whether a group pre‐existed outside of research or was research‐instantiated.

To wit, while we intentionally did not collect demographic information outside of the four V‐CES identity categories, the experts and facilitators engaged in this project were, by and large, young adults, most of whom appeared to have received some college‐level education and all were English speakers. The virtual nature of our study also favoured individuals with access to technology and consistent internet access. In addition, our study did not account for balancing the various intersecting identities that existed within our community groups, that is, differences in age, gender, income, intersectionality with other groups, or different identities within the same community umbrella (such as gay vs. transgender experts from the LGBTQ+ CES). At times, this resulted in misalignment stemming from vastly different experiences between experts in the same CES.

Although the objective of this PR effort was to enable community values and needs to steer the direction of the resulting toolkit, as mentioned, there were pre‐existing design constraints stemming from the original grant proposal. At times, these constraints came into tension with expert suggestions, for example, our project being predicated on the use of Nudge Theory, while experts expressed a desire for a more direct educational approach. It must be noted that the resulting solution was a *collaboration* with experts, but that the experts did not have full control over the design.

## 6 Conclusion

Conducting meaningful PR entails strategically mitigating the culturally‐embedded power dynamics between academic and community researchers via methodological choices. While traditional CES offer an effective framework for engagement at a single point within a study, there is a recognised benefit to including community input *throughout* a study [7, 14, 16]. This paper describes an approach to adapting CES for sustained engagement with the same community experts and facilitators over a 3‐year design project. Specific aspects of this approach, including retaining consistent individuals, in addition to mindful choices regarding identity, capacity‐building and more, cultivated trust and hence facilitated authentic community contributions that genuinely informed this study's outputs. We offer our experiences as a reference for others interested in optimising the impact of their research through effective PR.

## Author Contributions

**Shauntey Kalweit:** conceptualisation, methodology, formal analysis, investigation, writing – original draft preparation, writing – review and editing, visualisation, project administration. **Carly Marten:** conceptualisation, methodology, formal analysis, investigation, writing – original draft preparation, writing – review and editing, project administration. **Odia Kane:** conceptualisation, methodology, formal analysis, investigation, writing – original draft preparation, writing – review and editing. **Kalei Glozier:** conceptualisation, methodology, formal analysis, investigation, writing – original draft preparation, writing – review and editing. **David Andres:** conceptualisation, methodology, formal analysis, investigation, writing – original draft preparation, writing – review and editing. **Melissa Creary:** methodology, formal analysis, writing – original draft preparation, writing – review and editing, funding acquisition. **Maile M. Tualii:** methodology, formal analysis, writing – original draft preparation, writing – review and editing, funding acquisition. **Joon‐Ho Yu:** conceptualisation, methodology, formal analysis, writing – original draft preparation, writing – review and editing, funding acquisition. **Megan Doerr:** conceptualisation, methodology, validation, formal analysis, investigation, resources, writing – original draft preparation, writing – review and editing, supervision, project administration, funding acquisition.

## Ethics Statement

Community members were engaged as expert consultants and were privy to the entire development of the toolkit; they were not research subjects; therefore, their involvement was not subject to review by an IRB. The protocol for the piloting of the toolkit by researchers and oversight boards, who were themselves research subjects, was reviewed and approved (WIRB #2017037); participants received information sheets and provided verbal informed consent prior to participation.

## Conflicts of Interest

The authors declare no conflicts of interest.

## Supporting information

Revised_Appendix.

## Data Availability

This is a methods paper; additional details about the methods are available from the corresponding author upon request.
