# Supplementary material for: Cultivating Authentic Partnership: Cooperative Development of a Toolkit to Mitigate Group Harm in Biorepository‐Enabled Research
Source: Health Expect. 2026 Mar 18;29(2):e70635. doi: 10.1111/hex.70635 (PMC13080890; doi:10.1111/hex.70635)

# Appendix

1. Videos used to prepare community experts prior to CES: [Video 1](https://vimeo.com/835581141), [Video 2](https://vimeo.com/839842041), [Video 3](https://vimeo.com/844384129), [Video 4](https://vimeo.com/849307382)
2. [Guidebook used to prepare community experts for co-analysis](https://docs.google.com/document/d/1S3I40T0lvCAmov3tHSWWXFpswilAhwWkEDmU4c39MZ4/edit?tab=t.0#heading=h.wgwpueduqupe)
3. Examples of themes developed during one co-analysis session (screenshots from Miro board):


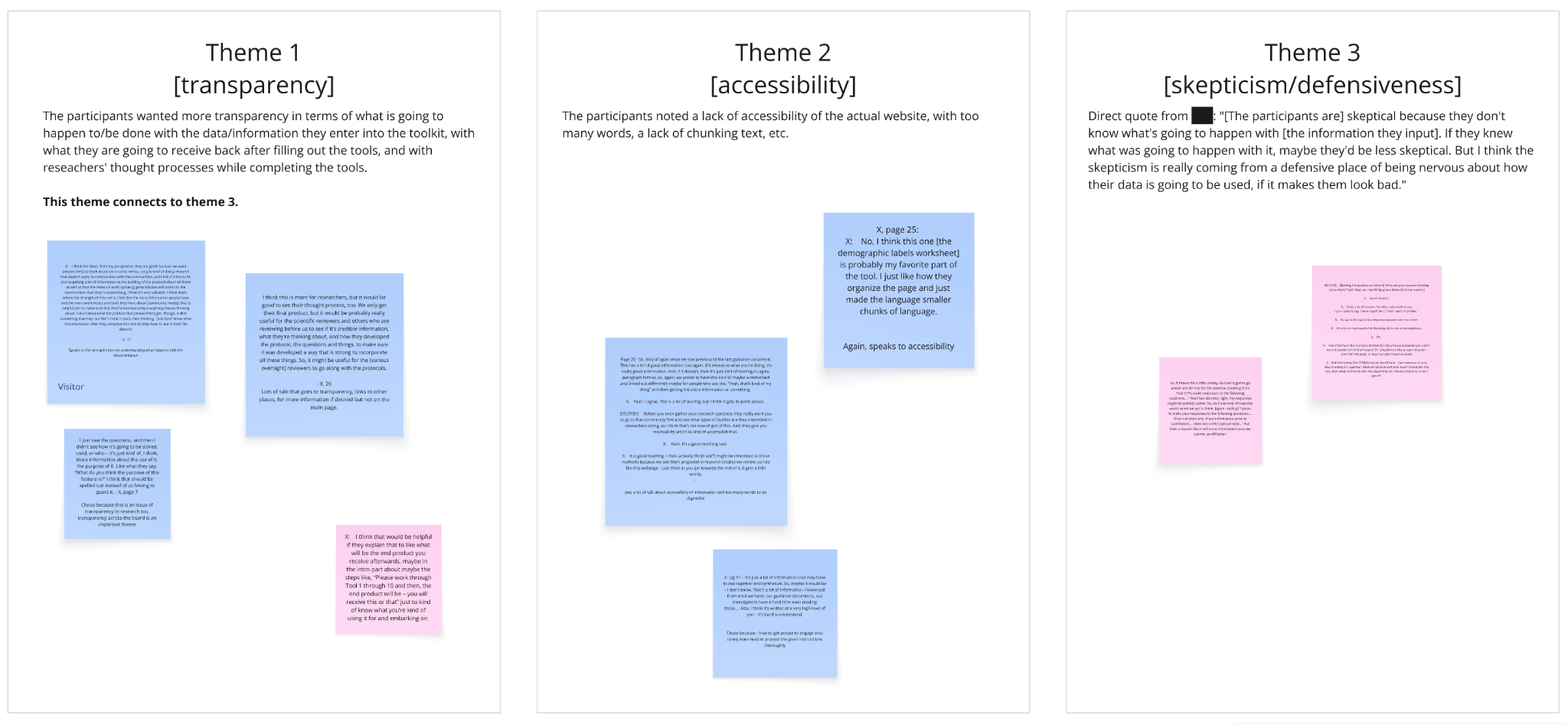


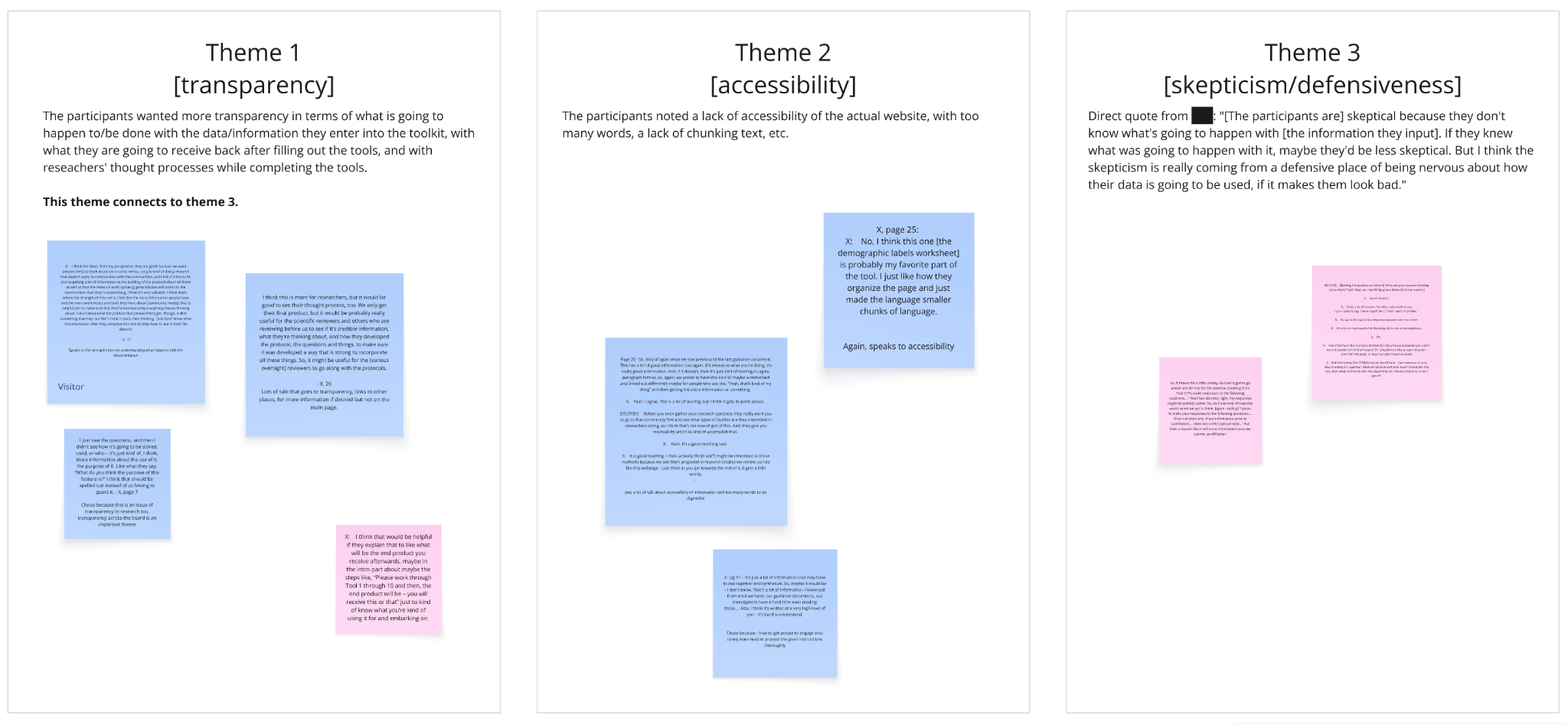


1. CHIRON homepage (screenshot):


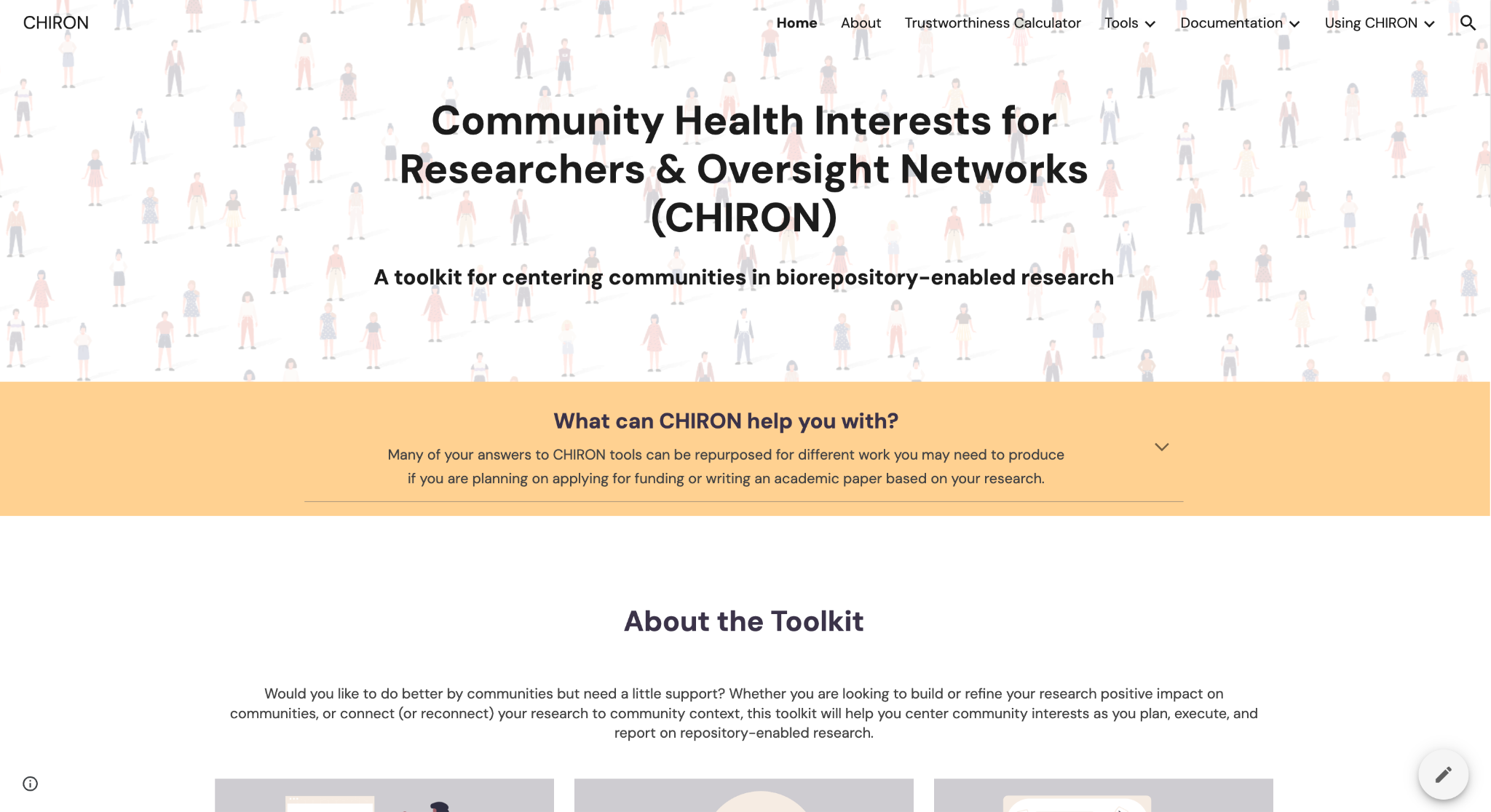

Supplement: Supplementary file 1 — Revised_Appendix. [file HEX-29-e70635-s001.docx]
